# Citizen science and niche modeling to track and forecast the expansion of the brown marmorated stinkbug *Halyomorpha halys* (Stål, 1855)

**DOI:** 10.1038/s41598-021-90378-1

**Published:** 2021-06-01

**Authors:** Jean-Claude Streito, Marguerite Chartois, Éric Pierre, François Dusoulier, Jean-Marc Armand, Jonathan Gaudin, Jean-Pierre Rossi

**Affiliations:** 1 UMR CBGP, INRAE, CIRAD, IRD, Montpellier SupAgro, Montpellier, France; 2 MNHN, Direction Générale Des Collections, Paris, France; 3 UMR SAVE INRAE Bordeaux Science Agro, ISVV, Bordeaux, France

**Keywords:** Climate-change ecology, Ecological modelling, Environmental social sciences

## Abstract

*Halyomorpha* halys (Stål, 1855), the Brown Marmorated StinkBug (BMSB) is a highly successful invasive species native to eastern Asia that managed to spread into North America and Europe in recent decades. We set up a citizen science survey to monitor BMSB expansion in France in 2012 and analyzed the data it yielded between 2012 and 2019 to examine the local expansion of the insect. These data were gathered with occurrences form various sources (GBIF, literature) to calibrate a species niche model and assess potential current BMSB range. We evaluated the potential changes to the BMSB range due to climate change by projecting the model according to 6 global circulation models (GCM) and the shared socio-economic pathways SSP245 in two time periods 2021–2040 and 2041–2060. Citizen science allowed to track BMSB expansion in France and provided information about its phenology and its habitat preferences. The model highlighted the potential for further range expansion in Europe and illustrated the impact of climate change. These results could help managing the current BMSB invasion and the framework of this survey could contribute to a better preparedness of phytosanitary authorities either for the BMSB or other invasive pests.

## Introduction

The number and the geographical distribution of non-indigenous species are increasing worldwide, with no sign of saturation^1^ and although cost estimation is difficult, these alien species are considered to be responsible for considerable environmental and economic losses worldwide^2^. The environmental impact of alien species is also expected to increase in the future as a consequence of climate change, human demography and international trade^3^. While environmental and socioeconomic impacts of biological invasions have surfaced as one of the hottest topics, biosecurity has emerged as a general concern^4^. One important tool of biosecurity is emergency preparedness and management. It encompasses efficient international coordination of early-warning systems, introduction prevention, effective response system to control and eradicate, sharing and access to reliable information among organizations for efficient surveillance and personnel training^4–8^. One key aspect of the preventive management of invasions is surveillance and early detection^7,9^. Crowd-sourced science has proven to be an effective approach to increased sampling coverage and early detection in a wide variety of life science disciplines^10–13^. Volunteers can prospect private spaces and often contribute through collecting data at a scale that is not allowed by conventional research organisms. Noticeably, crowdsourcing also acts as an educational tool contributing to improved public awareness, an important aspect to be considered with regards to future global systems of surveillance^7^.

An important aspect of preparedness is the knowledge of areas at risk likely to be invaded and how this risk might change in the future as an effect of the ongoing climate change. Climate suitability (CS) is a straightforward assessment of the potential distribution of a given species. It is not an assessment of the risk associated with a non-native species per se, but provides sensible information about the areas offering suitable conditions^14,15^. Species Distribution Models (SDM) have long been used to model species climatic niches and identify suitable areas outside the native range, thus providing interesting hints at where to increase surveillance and public information^16–18^. As such, SDM are central tools to official pest risk assessment^14^ and benefit from citizen science that provide large-scale comprehensive datasets needed to calibrate niche models^19,20^. Forecasting climate change relies on General Circulation Models (GCM) that use a system of mathematical equations capable of representing the movement of mass and energy across the atmosphere, the ocean and the lands and simulating the response of global climate to the increasing greenhouse gas emission^15,21^. Different GCM are available and choosing which ones to use has been shown to constitute a very important factor of SDM projection variability^22^, although it is often overlooked^23^. As a consequence, it is sensible practice to use more than one GCM to explore the uncertainty in possible future climates to improve the credibility of impact analysis^21^.

*Halyomorpha* halys (Stål, 1855), the brown marmorated stinkbug (BMSB) is a typical example of a successful invasive species. It is native to eastern Asia and is common in the temperate regions of China, Japan and the Republic of Korea^24^. In recent decades, the BMSB has spread outside its natural range: the first natural breeding populations of North America were reported in 2001, although it is considered to have been present since at least 1996. It has since colonized numerous states of the United States of America and Canada. A few years later, it was detected in Europe (2004) and has since expanded in more than 20 countries from Sweden to Greece and Spain to Turkey, the South European Territory of Russia (Krasnodar region) and Abkhazia^25,26^. The BMSB was recently detected in South America in Chile in 2017^27^. As with other successful invaders, the BMSB is highly polyphagous and has a vast range of more than 120 wild or cultivated host plant species^28^. The BMSB is responsible for important damage in agriculture in North America, while its harmfulness in European agriculture has only been reported recently^29,30^. As a highly polyphagous emerging pest worldwide, the potential geographical distribution of the BMSB has emerged as an issue of concern and several SDM have been established^31–33^. They all indicate that the range of the species is expected to expand in the near future, since not all suitable areas have yet been colonized.

In the present paper, we focused on the BMSB as a model species and we analyzed the data collected during two programs of citizen science initiated when the species started to spread in Europe and in France. We explored the validity of the data, the diversity of the volunteer profiles and the temporal dynamics of their contributions. The occurrence data were also used, together with additional data available from the Global Biodiversity Information Facility (GBIF) and the literature, to calibrate an SDM. This model is thus based on an up-to-date BMSB dataset and makes use of the newest current and future climate projections. The model predictions were used to identify suitable areas worldwide. Because preparedness calls for an assessment of the possible evolution of such categories according to climate change, we used the model to estimate the worldwide potential distribution of the BMSB according to 6 GCMs and the shared socio-economic pathways SSP245 in the period 2021–2040 and 2041–2060. In so doing, we obtained an assessment of the uncertainty that could be ascribed to GCM differences regarding the climate descriptors that drive the potential distribution of the species.

## Materials and methods

All the data analyses and graphical outputs were done using the R language^34^.

### Occurrence datasets

We collected occurrences from different sources: the international database GBIF (Global Biodiversity Information Facility: https://www.gbif.org/fr/), French citizen science campaigns supported by a smartphone application (Agiir by Inrae http://ephytia.inrae.fr/fr/P/128/Agiir and INPN-Espèces by MNHN: https://inpn.mnhn.fr/espece/jeudonnees/21092), citizen sightings and reports from entomological associations and naturalists, and data from the literature. These heterogeneous data were carefully examined in order to check the validity of the occurrences. For all sources, the diagnosis consisted of evaluating (1) the level of confidence in the taxonomic identification of *H. halys* and (2) the accuracy of the geographic localization. The validity of the record date was also checked in the case of citizen contribution. Additional information can be found in supplementary material and in Figure S1.

### Species distribution modelling

#### Model algorithm

Since we study an organism whose geographical distribution is rapidly changing because of its expansion, we are in a typical non-equilibrium situation. As a result, it is particularly difficult to obtain true absences; that is, to identify localities where BMSB is absent because of non-suitable environmental conditions rather than because of (transient) dispersal limitations^35,36^. For that reason, we used a presence-only algorithm and we retained Maxent^37–39^ because it has been shown to perform well, including in situations where the aim is to estimate habitat suitability in novel environments (i.e. future climate conditions)^40^. Maxent is a general-purpose machine learning method that uses presence points and a set of background locations that are not interpreted as absences, but rather provide information representing the range of environmental conditions across the study area^37,38^.

#### Environmental datasets

This study is based on climate data available from the worldclim database v2.1 (https://www.worldclim.org/)^41^. Worldclim provides both average climate values for the period ranging from 1970 to 2000 and yearly data from 2001 to 2018. The latter were averaged to obtain a dataset representative of the current conditions i.e. 2001–2018. Future climate projections described 2 periods spanning from 2020 to 2060 (2021–2040, 2041–2060). For each period, we considered 6 GCMs: BCC-CSM2-MR^42^, CNRM-CM6-1^43^, CNRM-ESM2-1^44^, CanESM5^45^, MIROC-ES2L^46^, MIROC6^47^ and the shared socio-economic pathways SSP245.

Shared Socioeconomic Pathways (SSPs) are scenarios of projected socioeconomic global changes used to infer greenhouse gas emissions according to various climate policies. We used SSP245 which is considered as a scenario of moderate future greenhouse gas emissions. It stabilizes radiative forcing at 4.5 W m^−2^ in the year 2100 corresponding to an increase of 1.1 °C to 2.6 °C of global mean surface temperature by the end of the twenty-first century^48^. The data used in the study corresponded to a resolution of 2.5 min (≈21 km^2^ at the equator).

Determining which climate descriptors are most informative and must be used to calibrate a niche model is both difficult and challenging^49^. We identified a set of climate variables that were a priori good candidates as they convey thermal constraints in different ways: maximum temperature of warmest month (bio5), minimum temperature of coldest month (bio6), mean temperature of wettest quarter (bio8), mean temperature of driest quarter (bio9), mean temperature of warmest quarter (bio10) and mean temperature of coldest quarter (bio11). Variables describing precipitations were not included in our analyses because the observed BMSB distribution may be partly affected by agricultural irrigation which cannot be accounted for in the available climate datasets. Some explanatory variables may be collinear with possible effects upon species distribution models. This point is discussed below.

#### Occurrence data thinning and background points

##### Occurrences

The model was calibrated using a subset of available occurrence data corresponding to the core distribution of the BMSB. We defined three core areas, i.e. Asia, North America and Europe (Supplementary Figure S2A). The rationale is that occurrences located at the margin of these areas, although valid, could correspond to unsettled populations or interceptions.

We did not distinguish between occurrences originating from native or invaded ranges because adaptive shifts occurring in the course of the process of the invasion may change the realized niche allowing invasive species to spread in areas where climate conditions change from the ones prevailing in the native range^17,50^. Data stemming from citizen science and more generally occurrences available for distribution modeling might be geographically biased if observers do not follow a coordinated spatial sampling plan. We addressed the problem by pre-processing the data by means of an environmental filtering step (details below). Such procedure is known to reduce the effects of sampling bias and improve model performances^51,52^.

##### Background data

Both the number of background locations and the area within which they are distributed are known to alter the outputs of the Maxent algorithm^53^. The most common way to distribute background points is random sampling. Amongst other available strategies^49^, we retained this procedure because it relies on the least assumptions about the species distribution and its relationships with environment^54^. We generated 10,000 randomly distributed background locations within the areas defined as core distribution areas (see Supplementary Figure S2B) excluding seas, lakes as well as locations where *H. halys* was present.

##### Environmental filtering

Our occurrence input dataset originates from heterogeneous sources and is subject to possible sampling bias which may alter model performances^51^. We addressed this issue by adopting an environmental filtering approach known to perform better than geographic filtering^52^. We performed a Principal Component Analysis (PCA)^55^ on the bioclimatic variables recorded at each occurrence point. We divided the range of the occurrence scores upon the first PCA axis in 50 bins. We used bins of similar amplitude to divide the range of the scores upon the second axis. The resulting grid was used to thin the occurrences on the basis of the following rule: when a grid cell contained more than one point, one point was randomly selected and retained for further analyses^56^.

#### Maxent calibration

The Maxent model was calibrated using the R package MIAmaxent^57^. It implements a flexible approach to Maxent calibration that allows to select a subset of transformed explanatory variables using a forward stepwise selection. This results in lower model complexity hence better model generalizability^58^, an interesting feature in the present study where we project model predictions outside of the spatial or temporal context of the data^59^. Five types of transformation were used for bioclimatic variables: linear, monotonous, deviation, forward hinge, reverse hinge and threshold^54,60^. For spline-type transformations (namely forward hinge, reverse hinge and threshold) an infinity of transformations is possible and the package MIAmaxent uses 20 for each and retain the ones that best explain the variation in the presence/background data (details in package documentation). The forward stepwise selection was carried out as a two-steps hierarchical procedure as detailed in^61^: first, a selection is performed amongst transformed variables derived from each explanatory variable. Second, explanatory variables represented by the transformed variables from first step explaining a significant amount of variation in the response variable under the significance threshold α = 0.001 are selected. At each step of the selection process, the fraction of deviance explained by the model is computed. It is analogous to the R^2^ of least squares regression^51^. Models are compared using a significance test accounting for sample size^61^.

Collinearity between explanatory variables is accounted for during the variable selection procedure as indicated in Vollering et al. 2019^57^.

#### Model evaluation

Model performance was assessed using the Continuous Boyce Index^62,63^ developed with the aim of evaluating presence-only models. In such situations, using conventional metrics such as the Area Under the Curve (AUC) of the receiver operating characteristic plot is problematic since they rely on true and false absences^64^. The CBI was presented by Hirzel et al.^63^ and Guisan et al.^49^. The range of the CS within the study area is partitioned into N classes. P corresponds to the frequency of occurrences falling into each class while E corresponds to the expected frequency of points randomly falling into each class. The P/E curve is the plot of P/E against the CS classes. Hirzel et al.^63^ indicate that good models lead to monotonically increasing P/E with increasing CS classes. The CBI is defined as the Spearman-rank correlation between P/E and CS classes, and it varies from + 1 (perfect prediction) to 0 (randomness) and -1 (counter-prediction).

In addition, the shape of the P/E curve indicates the suitability thresholds that can be used to reclassify CS maps into a set of several meaningful categories (see^63^ for details). Below the CS threshold at which P/E < 1, the model predicts fewer presences than expected by chance and CS values lower than this threshold correspond to unsuitable environments. On the contrary, CS values above the threshold at which P/E > 1 denote suitable conditions^63^. We also computed the AUC but this evaluation metrics should be interpreted with caution since it relies on absence locations and uses background points instead.

Ideally, the model should be evaluated using truly independent observations. In this study, we used the observations that were discarded during the data thinning process. These occurrences are valid and do not contribute to model calibration. A set of 34 datasets was constituted by randomly selecting 100 occurrence points (sampling without replacement) within the 3420 points discarded during the thinning process. We computed the 50% (median), 2.5% and 97.5% quantiles of the resulting P/E curves for each dataset. The CBI was computed as the Spearman-rank correlation between the values of the median and the values of the CS classes. The 2.5% and 97.5% quantiles were used to estimate CS thresholds following^63^.

#### Future geographical distribution

The model was projected using the data representing possible future climates for the period 2021–2040 and 2041–2060 and the SSP245. For each period, we computed the median of the predictions associated the different GCMs (BCC-CSM2-MR, CNRM-CM6-1, CNRM-ESM2-1, CanESM5, MIROC-ES2L, MIROC6) to derive a consensus model^49^. The resulting climate suitability maps were reclassified using the thresholds identified above for current climate conditions.

## Results

### Occurrence records and citizen science

A total of 9,467 occurrences were obtained from the GBIF database, of which 6,620 were considered to be valid (69.9%). Because citizen sightings were analyzed year by year, 2020 data were incomplete at the time of data analysis (mid-2020) and were thus discarded. It should be noted that citizen reports could be valid according to one criterion e.g. species identification whilst invalid for another e.g. missing date. As a consequence, the number of occurrences available could be different according to the question at hand.

A total of 3560 online forms were gathered by the Agiir application including 887 valid BMSB identifications (24.9%)^85^. The naturalist application INPN-Espèces gathered 147 valid occurrence data. Additionally, FD and JCS respectively gathered 74 and 87 reports from naturalist networks. Correct identification of *H. halys* accounted for 73% of the naturalist records gathered by JCS. Furthermore, JCS directly collected 377 reports from citizens off which 186 corresponded to valid identification of the BMSB. An additional 383 occurrences in Asia were gathered from Zhu^31^ and 17 supplementary occurrences were mined in the literature^26,27,65–68^.

**Figure 1:**
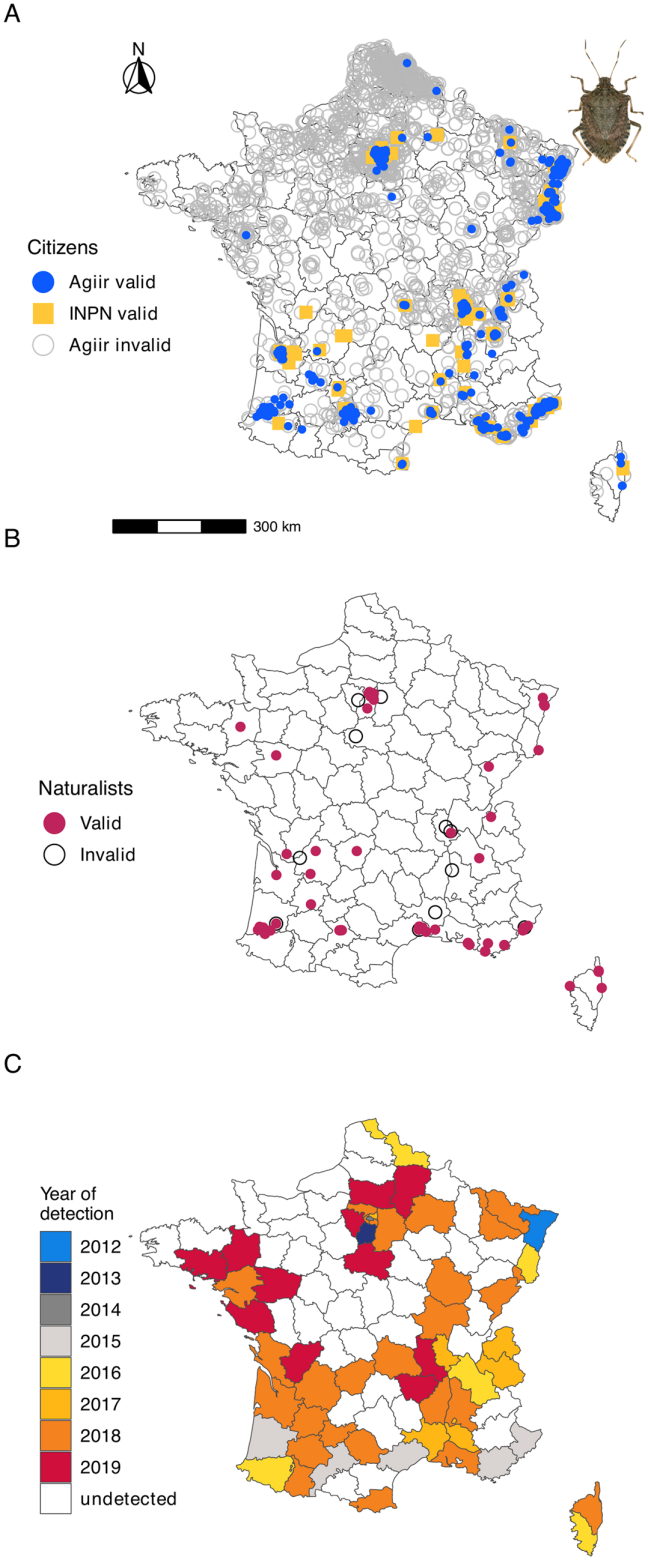
Occurrence data for *Halyomorpha halys* in France according to citizen sources. (**A**) Distribution of citizen contribution according to the French networks Agiir (Inrae) and INPN-Espèces (MNHN). (**B**) Distribution of sightings from naturalist sources. (**C**) Geographical expansion of the BMSB in France from 2012 to 2019 according to available data including GBIF sources. Photograph by J.C. Streito/Inrae. The maps were generated using R 4.0.3 (https://cran.r-project.org/).

Figure 1A shows the geographical distribution of both Agiir and INPN-Espèces citizen networks. Overall, sightings are somewhat homogeneously distributed, although valid records are clearly spatially aggregated and indicate the clusters of colonized areas. Naturalist sightings are less numerous, but the proportion of valid records is very high compared to citizen contributions (Fig. 1B). Figure 1C shows the dynamics of the BMSB expansion across French departments as depicted by citizen science data and GBIF records.

**Figure 2:**
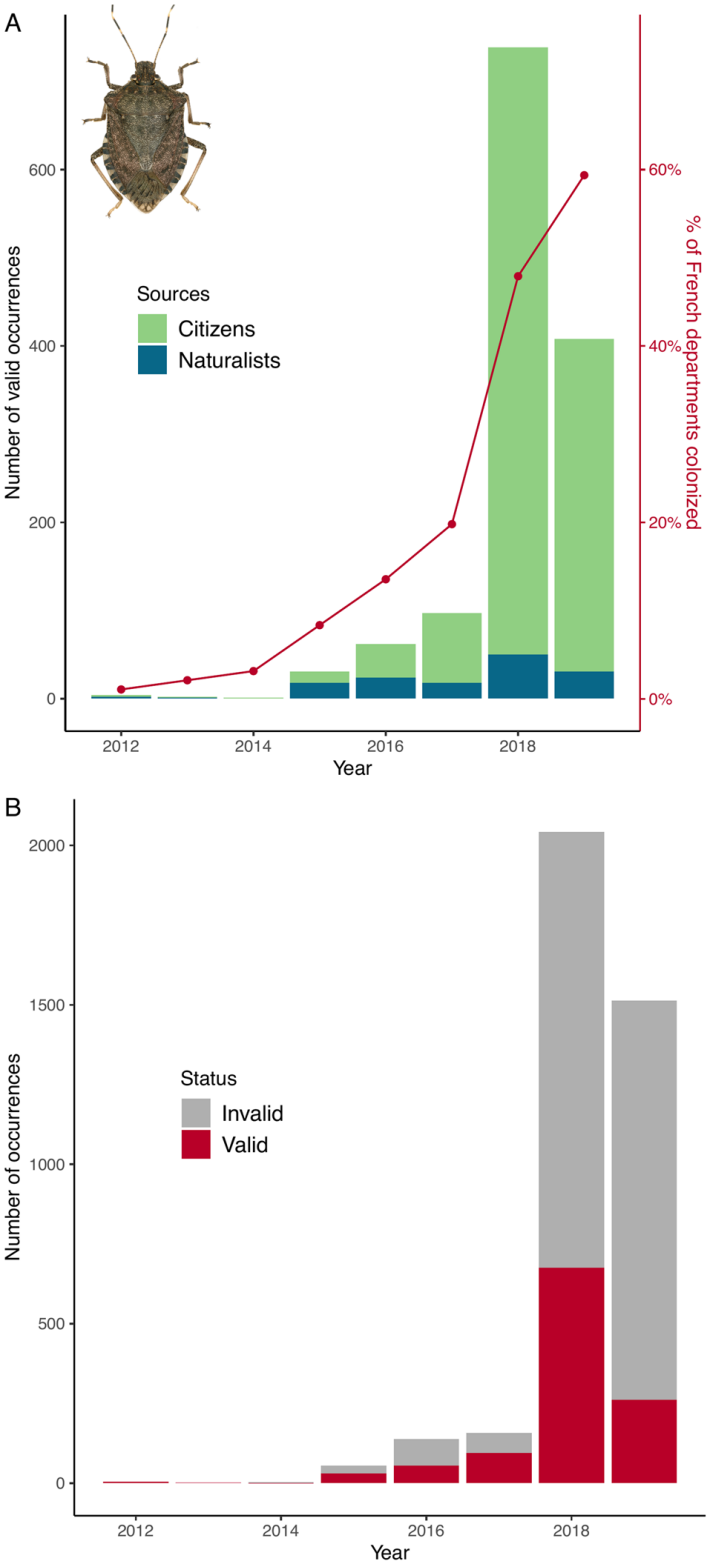
Temporal dynamics of the brown marmorated stinkbug *H. halys* in France as revealed by citizen contribution. (**A**) Changes in the number of valid sightings over 8 years of the study. BMSB expansion in France is illustrated by the proportion of departments colonized. (**B**) Evolution of the total number of sightings (Agiir and INPN-Espèces plus additional records from naturalists). Photograph by J.C. Streito/Inrae.

Figure 2A shows the evolution of the total number of valid sightings either from citizens or naturalists. The number of records from both networks decreased in 2019 but remained much larger than in 2012–2017, and 11 French departments were newly colonized that year (Fig. 1C). Both the number of valid and invalid sightings increased in 2018 and 2019 while the ratio of valid records decreased (Fig. 2B).

**Figure 3:**
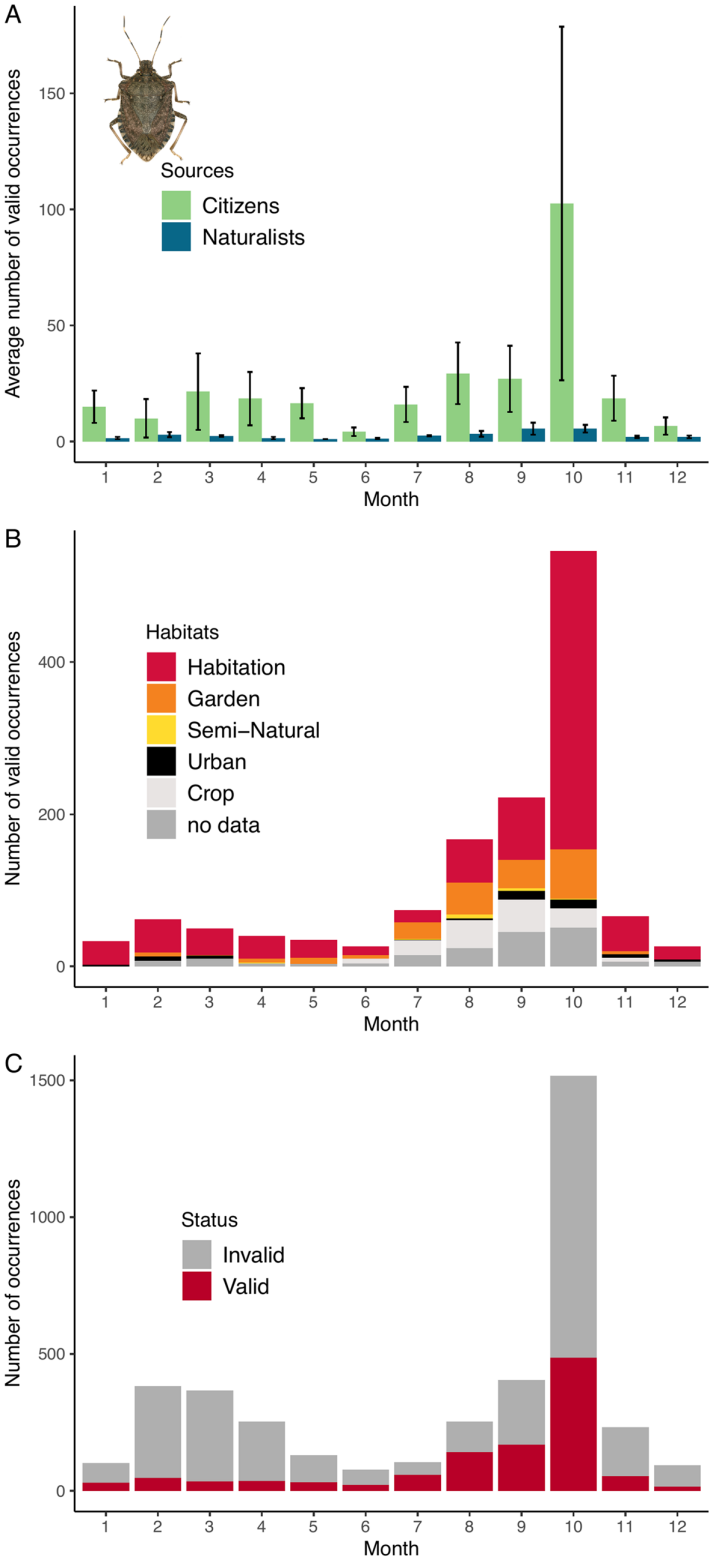
Phenology of the brown marmorated stinkbug *H. halys* sightings in France. (**A**) Changes in the monthly average number of valid occurrences (2012–2019). Error bars depict the standard error *i.e.* standard deviation of the mean. (**B**) Changes in the monthly average number of valid occurrences according to the habitat where observation was made (see text for details). (**C**) Changes in the monthly average number of occurrences (2012–2019). Photograph by J.C. Streito/Inrae.

Figure 3A shows the monthly number of valid records (averaged through the years) from Agiir, INPN and the naturalist network. A marked increase occurs in October which corresponds to the period where BMSB starts to enter houses in search for an overwintering site. Overall, in France and since 2012, the BMSB has largely been reported in urban environments (streets, parks, greenings) and in houses and remained rare in semi-natural areas (Fig. 3B). So far, the species has caused limited crop damage in France. If the number of sightings increases at the beginning of autumn (Fig. 3C), it concerns both valid and invalid records, possibly due to various species entering houses including other pentatomids such as the mottled shieldbug *Rhaphigaster nebulosa* that is commonly mistaken for the BMSB in France^69^. Other pentatomids reported are: *Nezara viridula* (L., 1758), *Dolycoris baccarum* (L., 1758), *Palomena prasina* (L., 1760) and *Pentatoma rufipes* (L., 1758). *Leptoglossus occidentalis* Heidemann, 1910 (Coreidae) is also frequently signaled.

### Occurrence records for niche modeling

A total of 8,387 points corresponding to valid BMSB identification was available for modelling. Points falling inside the same pixel of the climate raster, points falling into oceans, points with missing date and points located outside the core areas shown in Figure S2A and S2B were discarded, leading to a total of 4144 occurrences. Environmental thinning resulted in removing 3420 occurrences leading to a final dataset of 724 points (17.47%).

### Models calibration and evaluation

The 6 original climate variables (bio5, bio6, bio8, bio9, bio10, bio11) used as explanatory variables were transformed into 52 derived variables among which 5 were finally selected by the forward procedure. These transformed variables included 3 deviation-type transformations with parameters of 2, 0.5 and 2 for bio5, bio8 and bio10 respectively, the variable bio6 after a reverse hinge transformation with a knot value of 7 and the variable bio8 after a threshold transformation for a knot value of 9. Details can be found in Vollering et al. 2019^57^ and Phillips and Dudík 2008^54^.

The model was evaluated using the 3,420 occurrences data that were excluded from the calibration dataset during the thinning process. The P/E curve showed an increasing shape that indicated a well performing model (Figure S3). The CBI was 0.97 which denoted the good performance of the model. The AUC was 0.838. The median and its lower and upper bounds (quantiles) are shown in supplementary Figure S3. Two thresholds could be identified (th1 = 0.244, th2 = 0.49) respectively corresponding to the CS values for which the upper and the lower bounds reached a P/E > 1. These thresholds were subsequently used to reclassify the CS map as follows: CS < th1 corresponded to unsuitable climate conditions; th1 ≤ CS < th2 corresponded to marginal climate conditions and CS ≥ th2 indicated suitable climate conditions. We here considered the marginal class to represent climate suitability slightly higher than 1 in a large proportion of randomizations. This could be interpreted as areas where climate conditions are near limiting values such as what could be expected at the margin of the core geographical distributions. In some cases, spill-over from neighboring suitable sites could maintain local populations despite barely favorable conditions.

### Climate suitability map

**Figure 4:**
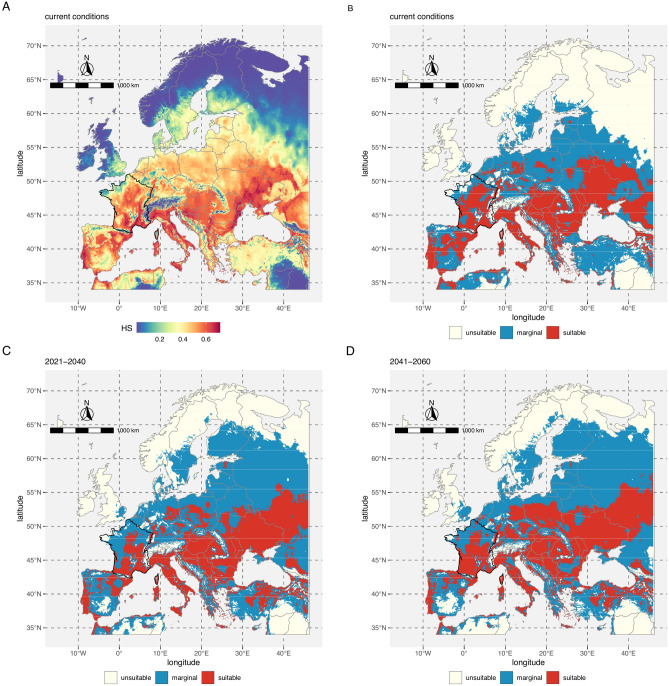
Potential geographical distribution of the brown marmorated stinkbug *H. halys*. (**A**) Climate suitability estimated by a maxent model according to current climate conditions. (**B**) Reclassified climate suitability according to current climate conditions. (**C**) Reclassified climate suitability for the period 2021–2040. The underlying climate suitability is a consensus derived for the projections of the model with 6 global circulation models. (**D**) Reclassified climate suitability for the period 2041–2060. The underlying climate suitability is a consensus derived for the projections of the model with 6 global circulation models and the shared socio-economic pathways SSP245. The maps were generated using R 4.0.3 (https://cran.r-project.org/).

Figure 4A shows the Maxent logistic estimation of the climate suitability in Europe according to current climate conditions. The reclassified suitability map for current conditions is shown in Fig. 4B. Figure 4C,D illustrate the reclassified predictions for future climate conditions in the period 2021–2040 and 2041–2060 respectively. Each future projection corresponded to a consensus model computed from the predictions associated to the 6 GCMs used in the study. Maps showing the worldwide estimations are given in supplementary material (Figures S4 to S9).

**Figure 5:**
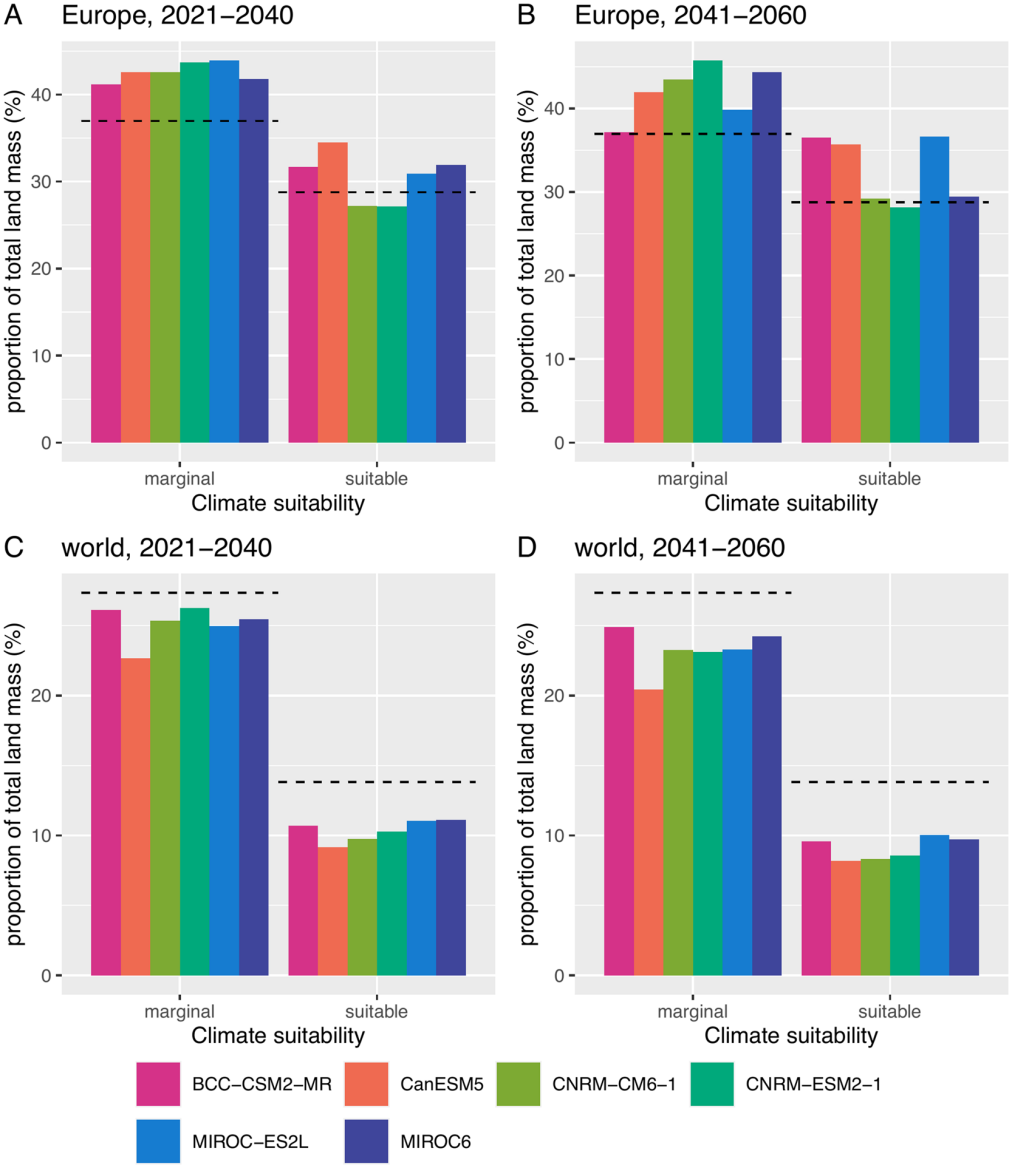
Proportion of total land mass expressed in percentage that are predicted as marginal or suitable according to 6 global circulation models and the shared socio-economic pathways SSP245. (**A**) Estimates for Europe in 2021–2040. (**B**) Estimates for Europe in 2041–2060. (**C**) Worldwide estimation in 2021–2040. (**D**) Worldwide estimation in 2041–2060. Dashed lines indicate the proportion of total land mass for current climate conditions. Europe is delimited by longitudes -15 and 46 and latitudes 34 and 72 decimal degrees.

Figure 5 shows the proportion of land surfaces that fall into marginal or suitable categories in the periods 2021–2040 and 2041–2060 according to the SSP245 in Europe and in the world. At the scale of the world, both the proportion of marginal and suitable areas are expected to decrease in the 2021–2060 and 2041–2060 periods. This pattern is true whatever the GCM considered. However, in Europe, this trend was not observed and an increase in marginal and suitable conditions at the expense of unsuitable conditions is expected according to most of the GCMs. These results indicated that climate change will lead to harsher worldwide climate conditions for BMSB while a different situation would occur in Europe where better conditions are to be expected.

## Discussion

Our results show that citizen science provides valuable data allowing us to characterize the spread of *H. halys* as well as understand some important aspects of its biology and track its spread in France. In a recent study, Maistrello et al.^12,70^ came to a similar conclusion in different regions of Italy. Stoeckli et al.^71^ used records collected in a citizen science initiative to assess the potential for BMSB spread in Switzerland 15 years after its introduction and showed that nearly all suitable areas have been colonized. In France, the spread has accelerated in recent years, with more than 50% of departments colonized between 2018 and 2019. Interestingly, the volunteers differed in their accuracy of species identification; naturalists were better at identifying the BMSB, while citizens were prone to confusing it with other insect species such as native stink bugs. However, the large number of records stemming from non-naturalists compensated for their limited identification skills and led to a large number of valid sightings in various regions of the country. It must be noted that these data come at the expense of the important work of sorting and checking the records stored in our databases, highlighting the fact that citizen science should not be considered as a ‘magic bullet’.

Another interesting outcome of the survey is the possibility of gathering information on the phenology of the species. This has already been illustrated in the seminal paper by Maistrello et al.^70^ where the authors reported a peak in sightings in October in the Emilia Romagna region (Italy). We reported a similar peak in our study. This pattern is likely to reflect the well-known behavior of the BMSB in which it tends to enter houses and human installations in the fall to find shelter and then overwinter^24^. Furthermore, several records indicate that BMSB is attracted to domestic light, which probably reinforces their taste for buildings^72^. The October peak also coincided with a marked increase in sightings recorded in various habitats that were gathered under the name “houses” (home, flat, buildings). Overall, the number of sightings increased in the period spanning from August to October. Observations in agricultural areas and urban environments (gardens, parks, streets) occurring during that period may indicate increased activity at the end of summer and in early fall as pre-overwintering activity. It is worth noting that although there were more reports in October, it did not yield an increase in valid sightings. The increase in reports included numerous misidentifications with pentatomids, such as *Rhaphigaster nebulosa*, *Nezara viridula, Dolycoris baccarum* or with the invasive Coreidae *Leptoglossus occidentalis*, which have similar behavior and enter human-made structures at the same time of the year. In fact, non-naturalist citizens tend to report true bugs they observe in their homes or gardens without really considering whether it is BMSB or not. In addition, a number of citizens report the insects they observe knowing that they are not the proper species. These forms increase the validation effort, but also provide opportunities to monitor non-target invasive species that could be detected earlier. An interesting aspect of citizen contribution is that it potentially offers information on phenology and behavior within anthropic habitats (e.g. semi-rural habitats, urban areas) where typical agricultural pests are seldom surveyed. Beyond the specific context of agricultural threats, such data could be useful to manage the BMSB as a domestic pest, namely in an urban context where it is considered a nuisance^72–75^.

Because of its behavior and its rather large size, the BMSB is a good example of how citizen science can contribute to studying agricultural pest invasions. Once refined and checked, the datasets may be used to calibrate expansion models and possibly to monitor population dynamics in various part of the country. Nevertheless, our results show that it takes several years (2012–2015) for the number of valid sightings to reach more than 10 per year. This reflects the fact that (1) at the beginning of the invasion, the insect density was low and (2) that volunteers were not well aware of the BMSB. This is mostly true for non-naturalist contributors, while naturalists, although much less numerous, contributed to the valid sightings in the early stages of the invasion. In the specific context of the BMSB expansion in France, we informed citizens as early as possible through various channels including national publications (in French)^69^ and continue to do so^76^. The initiation of a system that led to a regular flow of occurrence data took several years. As such, citizen science did not allow for a significant early warning system per se. However, it did help in monitoring the BMSB expansion after approximately four years following the launch of the campaign. We postulate that this appears to be the amount of time needed to either educate and make people aware of the BMSB problem, and/or for the insect population to reach density levels where its inconvenience generates public attention and willingness to contribute to citizen science projects.

We can consider three main phases in the BMSB invasion during which different actors can be mobilized for monitoring (1) at the very beginning of the invasion, when the populations of the invasive species are low, naturalists and official monitoring services who are excellent observers always on the look-out for novelties and are highly effective in detecting insects that are still rare and difficult to observe (2) when abundance increases, non-naturalist citizens relay and by their much higher number provide information on the dynamics of the invasion and indirectly on the level of populations (3) when populations become large enough, agricultural professionals take over and can accurately assess the levels of population and damage. The growing number of reports in the fall in homes starting in 2017 is a warning of an increase in BMSB populations. This alert is important from an agronomical point of view because this species is much more difficult to detect and observe in crops and natural environments. In 2017, although the number of bugs exploded in homes, they were still virtually undetected by agricultural professionals, yet extremely mobilized. Although most citizens' sightings are recorded in residential districts, they are scattered across the whole country including rural areas where the presence of the BMSB represents a risk for agriculture.

The worldwide expansion of *H. halys* has sparkled a great scientific interest for its potential range expansion. Initiated by Zhu et al.^31^, various niche models have been developed to assess the areas where climate is suitable for the species^32^,see also^77,78^. Interestingly, both correlative SDM^31,77,78^ and the Climex mechanistic niche model^32,33^ are globally in agreement and indicate potential further expansion in Europe and north America. While correlative models such as Maxent seek for a link between species distribution records and environmental data, mechanistic bioclimatic models such as Climex focus on species ecophysiological responses to environmental variables^79^.

Our model showed than eastern European countries such as Poland, Czech Republic, Slovakia, Hungary, Romania, Bulgaria, Moldova, Belarus or Ukraine are at high risk of invasion. Some of these countries have already reported sightings and the further expansion is very likely. Non-colonized areas exhibiting suitable climatic conditions are at high risk of being invaded because dispersal limitations are low for the BMSB. Indeed, hitchhiker pests such as *H. halys* are mainly driven by human-mediated dispersal as the bug is hidden in various transported materials such as luggage, shipping containers, cars, trains or aircraft. One factor limiting phytophagous invasive species establishment is the availability of suitable host plants. In the case of the BMSB, this constraint is strongly relaxed since the insect is able to feed on a vast array of plants, comprising at least 120 wild or cultivated species^24^. Beyond the question of the establishment of the species is whether it would be responsible for important economic damage or not. In the US, the damage is very important^80^, while in Europe the first fruit injury and crop losses have been reported on pear and peach orchards in Italy^29,30^. Elsewhere in Europe, the BMSB is mainly a domestic inconvenience so far. In a recent study, Stoeckli et al.^71^ showed that both the number of generations per year and the activity periods in spring and late autumn could possibly increase in Switzerland as a result of climate change. Such changes in population dynamics or phenology could trigger more crop losses not only in Switzerland but in many European regions. This raises the issue of the climate change impact upon the future range of the BMSB.

According to our model, the range of the BMSB is expected to change in the coming decades as a consequence of ongoing climate change due to anthropogenic activities. Our projections show that the proportion of unsuitable land is expected to increase worldwide at the expense of suitable or marginal areas irrespective of the global circulation models considered. Interestingly, this pattern is not observed equally in all regions of the world. In Europe, the surface of suitable or marginal conditions is expected to increase in regions such as the United Kingdom, parts of Scandinavia, Finland, Lithuania, Latvia, Estonia and Russia. These trends are in line with the estimations of Kistner et al.^33^ who used Climex and two GCMs. On the other hand, Southern parts of the continent such as south Spain (part of Andalucia, Castiulle La Mancha, Extremadura, Región de Murcia) are expected to become unsuitable due to temperature increase. Suitability would also decrease in eastern Greece albeit to a lesser extent.

Although consistent, the predictions associated with each GCM varied, thus illustrating a well-known source of uncertainty^22,81^. GCM differ in the way they associate ocean, land and atmospheric fluxes of energy. In that way, they are all different, but it is not straightforward to assess the way these differences alter the outputs of the SDM combining more or less complex relationships between several bioclimatic variables.

There is a great deal of scientific evidence supporting the impact of global changes upon species geographical distribution. Matching climate velocity requires sufficient dispersal capacities, and not all species are likely to track the rate of environmental change^82^. Unlike some plants, insects with high dispersal potential such as the BMSB are very likely to be able to cope with climate velocity^83,84^. Adaptation is another process by which species can cope with environmental change, but ecological niche models are based on the assumption of niche conservatism^85^ and their outputs should thus be taken with caution in the context of climate change. In particular, areas considered to become unsuitable in the future may not always lead to range contraction if the BMSB could adapt to these changes. We do not have any clue as to how the BMSB could adapt to future climate conditions and thus future range predictions should be taken with caution, keeping in mind that they could be underestimated. Our model, and more generally all SDM are associated to a certain level of uncertainty chiefly because they assume that species are at equilibrium with the environment^59^. However, model predictions constitute valuable basis for discussion on areas at risk. One possible future action could be to set-up large scale and long-term citizen science initiatives along with improved information of authorities in areas at risk so as to improve preparedness through better surveillance and early detection.

## Conclusions/highlights

Early detection and tracking could efficiently include a citizen science component. Its deployment requires a medium- or long-term investment and an important volume of expertise from expert naturalists to process and check sightings. The behavior of the BMSB makes its sighting by non-expert people easy in autumn but, in turn, may lead to an urban bias. Citizen science provided consistent data on BMSB expansion, hosts, habitats and phenology. Species distribution models strengthen risk evaluation, surveillance, the overall preparedness of authorities and *in fine* policy. Either estimating current or future conditions, such tools are precious to help build efficient surveillance and set up early detection programs. Climatically suitable areas for the BMSB are not yet occupied and will expand in Europe in the future. Apart from Europe, suitable areas will contract in Asia, North America and in other parts of the world. Using various GCM allowed us to compute a consensus model that accounts for uncertainty associated with the different climate models.

## Supplementary information

Supplementary Information.

## Data Availability

Citizen data are available upon request to the authors. Other occurrences data are available from GBIF.org.
