# Supplementary material for: Citizen science and niche modeling to track and forecast the expansion of the brown marmorated stinkbug Halyomorpha halys (Stål, 1855)
Source: Sci Rep. 2021 Jun 1;11:11421. doi: 10.1038/s41598-021-90378-1 (PMC8169697; doi:10.1038/s41598-021-90378-1)
Supplement: Supplementary file 1 — Supplementary Information. [file 41598_2021_90378_MOESM1_ESM.pdf]

Citizen science and niche modeling to track and forecast the expansion of the brown marmorated stinkbug *Halyomorpha halys* (Stål, 1855)

Jean-Claude Streito[1], Marguerite Chartois[1], Éric Pierre[1], François Dusoulier[2], Jean-Marc Armand[3], Jonathan Gaudin[3], Jean-Pierre Rossi[1]

[1] CBGP, INRAE, CIRAD, IRD, Montpellier SupAgro, Montpellier, France

[2] MNHN, Direction générale des Collections, Paris, France

[3] SAVE INRAE Bordeaux Science Agro, ISVV, Bordeaux, France

Contents

1 Diagnosis of occurrence data **1**
1.1 Diagnosis of occurrence data from gbif.org . . . 1
1.2 Diagnosis of occurrence data from citizen science . . . 1
1.2.1 Agiir . . . 1
1.2.2 INPN-Espèces . . . 1
1.2.3 Naturalists reports and additional citizen sightings . . . 2
1.3 Records collected from the literature . . . 2
1.4 References . . . 2

2 Figures **3**

1 Diagnosis of occurrence data

1.1 Diagnosis of occurrence data from gbif.org

We used the R package `rgbif`[1] to retrieve data from the GBIF system. All occurrences labelled “invalid basis of record” or associated with missing or $(0, 0)$ spatial coordinates were discarded.

1.2 Diagnosis of occurrence data from citizen science

1.2.1 Agiir

The Agiir application (details are available at: http://ephytia.inrae.fr/fr/P/128/Agiir) allows volunteers to report their real-time sightings as forms containing pictures and comments. Date and geographical coordinates are automatically added to the form on the basis of the smartphone GPS localization. We analyzed all reports from 15/08/2012 to the end of 2019[1] and checked species identification on the basis of uploaded pictures. Sightings with no or poor quality picture were considered “invalid”. Taxonomic characters used for specimen identification are illustrated in Figure S1. Occurrences from Agiir falling in the sea and/or in countries far away from the known *H. halys* range (e.g. Niger, Benin, Cameroun) and were discarded. Reports from previously unoccupied regions were carefully checked and observers were occasionally contacted to supplement information.

1.2.2 INPN-Espèces

INPN-Espèces application (details available at: https://inpn.mnhn.fr/informations/inpn-especes) allows citizens to report their biodiversity observations. Each observation must be uploaded with one to three pictures used for identification by MNHN teams. As for the Agiir application, date and geographical coordinates are automatically recorded through the GPS localization of the smartphone. The occurrence data extracted covered the period from 15/08/2012 to the end of 2019. All Heteroptera occurrences were checked and valid occurrence records

[1]Data collection is still under way

DOI: 10.1038/s41598-021-90378-1

were disclosed and distributed within the framework of the national database of natural heritage (https://inpn.mnhn.fr/).

1.2.3 Naturalists reports and additional citizen sightings

In contrast to citizens who are volunteers with no special a priori knowledge, we defined a second class of contributors named “naturalists”. Naturalists are people with amateur or confirmed knowledge in entomology who directly report their observations (phone, emails) or use entomologist networks such as *Le monde des insectes* (www.insecte.org). JCS collected sightings from naturalist sources (from 15/08/2012 to the end of 2019) and checked the species identification. FD gathered similar information from 21/08/2015 to 28/03/2020. Additional citizen sightings directly reported, outside the Agiir and INPN-Espèces applications, were also collected by JCS (from 01/06/2015 to the end of 2019). Records with missing dates, missing pictures or specimens, poor picture definition or missing spatial coordinates were discarded. When spatial coordinates were missing and the report was associated to precise information about the locality where the observation had been made, the centroid of the locality served as sighting location.

1.3 Records collected from the literature

We added the occurrence data from the literature documenting the distribution of the BMSB in its native range[3] and in various recently colonized areas.

1.4 References

[1] Chamberlain S., Barve V., Mcglinn D., Oldoni D., Desmet P., Geffert L. & Ram K. 2020. `rgbif`: Interface to the Global Biodiversity Information Facility API. R package version 3.4.0, https://CRAN.R-project.org/package=rgbif.
[2] Hudecheck M. 2017. `revgeo`: Reverse Geocoding with the Photon Geocoder for OpenStreetMap, Google Maps, and Bing. R package version 0.15. https://CRAN.R-project.org/package=revgeo
[3] Zhu, G., Bu, W., Gao, Y. & Liu, G. 2012. Potential Geographic Distribution of Brown Marmorated Stink Bug Invasion (*Halyomorpha halys)*. PLoS ONE 7, e31246.

DOI: 10.1038/s41598-021-90378-1

2 Figures

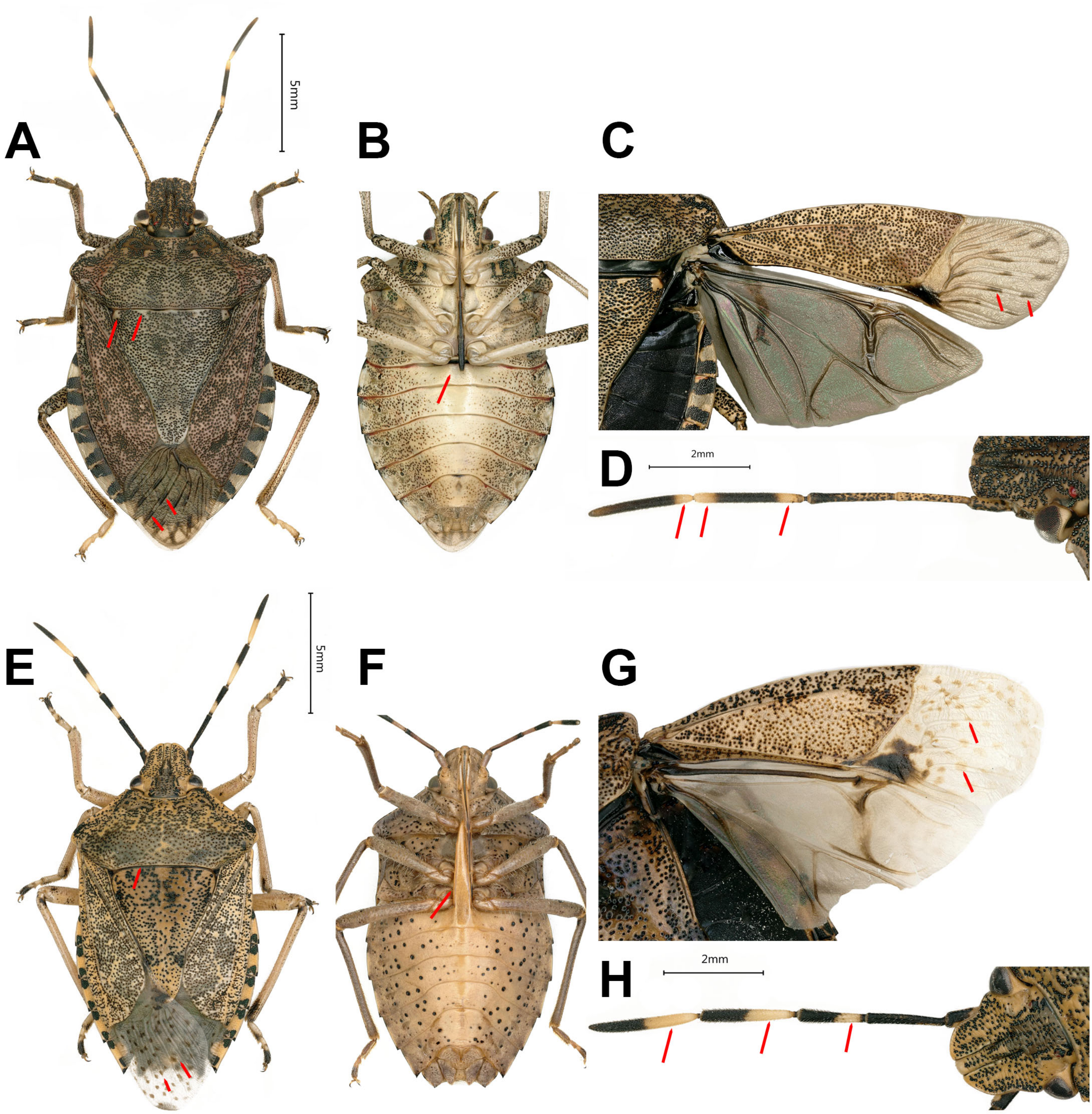

Figure S1: Morphological characters used to distinguish the brown marmorated stink bug *Halyomorpha halys* from *Rhaphigaster nebulosa*. **A-D**: *H. halys*. **A**: habitus upperside (arrows: light calus on scutellum base and elongated dark marks on the membranes). **B**: underside (arrow: no spine on the base of abdomen). **C**: wings (arrows: elongated dark marks on the membrane). **D**: antennae (arrows: position of the white rings). **E-H**: *R. nebulosa*. **E**: habitus upperside (arrows: no light calus on scutellum base and rounded dark marks on the membranes). **F**: underside (arrow: spine on the base of abdomen) **G**: wings (arrows: rounded dark marks on the membrane). **H**: antennae (arrows: position of the white rings). Sources: J.C. Streito/INRAE.

DOI: 10.1038/s41598-021-90378-1

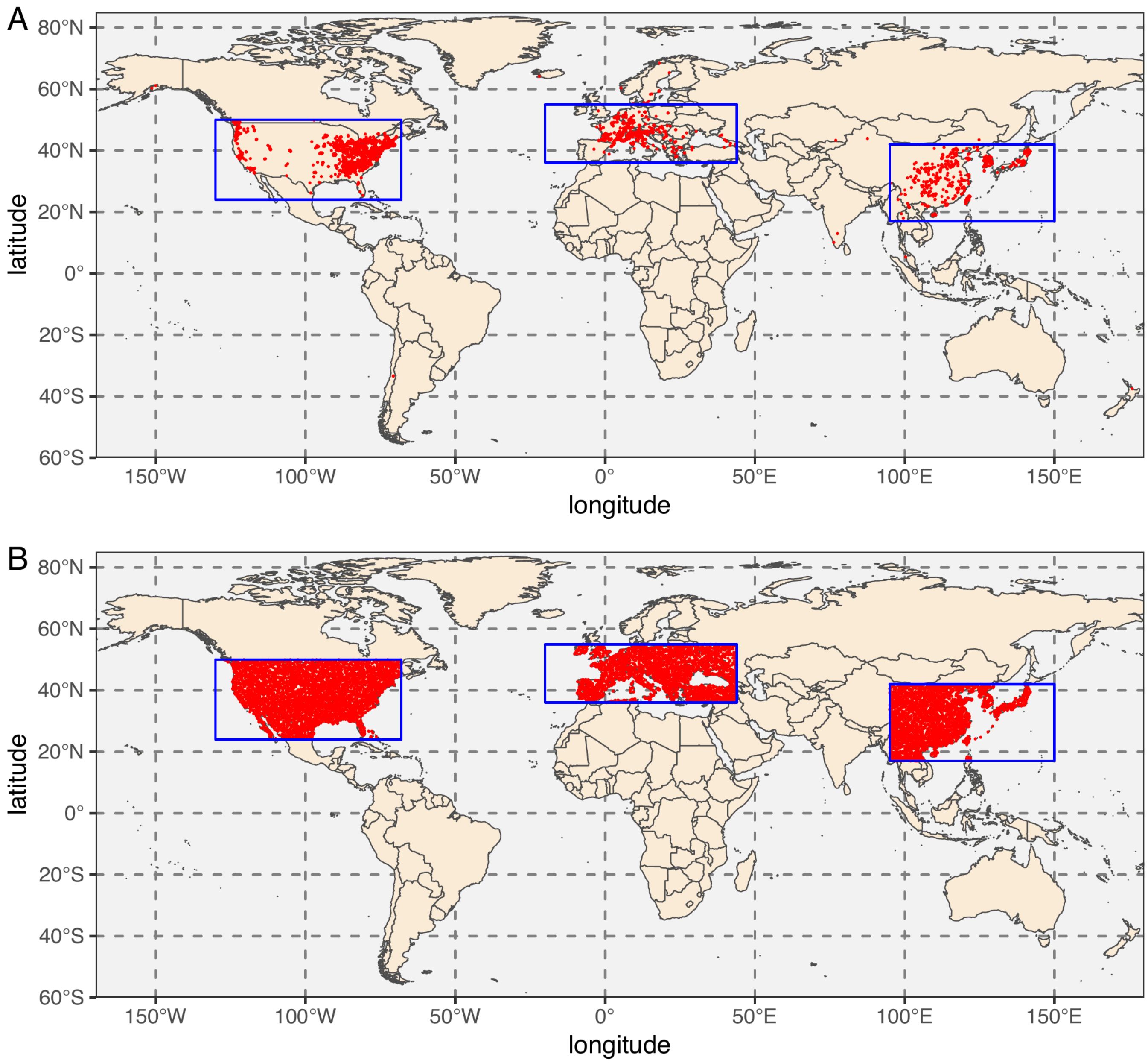

Figure S2: Distribution of occurrences and background points used to model the potential geographical distribution of the brown marmorated stink bug *Halyomorpha halys*. **A**: Worldwide distribution of valid occurrence data for *H. halys*. Data include citizen networks, literature and GBIF sources. Blue rectangles indicate the limit of the core areas where breeding populations of BSMB are assumed to be present. Occurrences located outside core areas were discarded from model calibration. **B**: Distribution of the 10,000 background points used in the Maxent model calibration. The maps were generated using R 4.0.3 (https://cran.r-project.org/).

DOI: 10.1038/s41598-021-90378-1

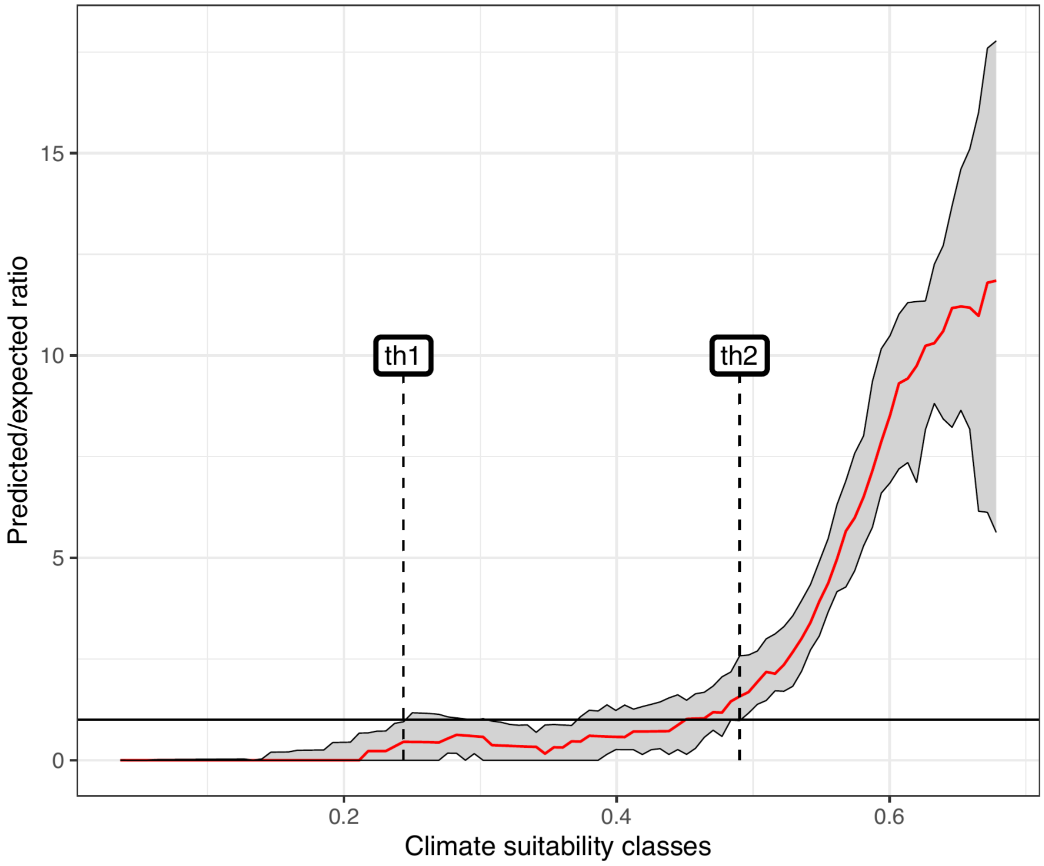

Figure S3: The Continuous Boyce Index. Changes of the predicted/expected curve with increasing climate suitability. Upper and lower bounds are respectively 2.5% and 97.5% quantiles derived from 34 replicates (see text for details). The curves are used to define the thresholds th1 and th2 of climate suitability (Hirzel et al. 2006). The line defined for predicted/expected ration = 1 corresponds the output of a random model.

DOI: 10.1038/s41598-021-90378-1

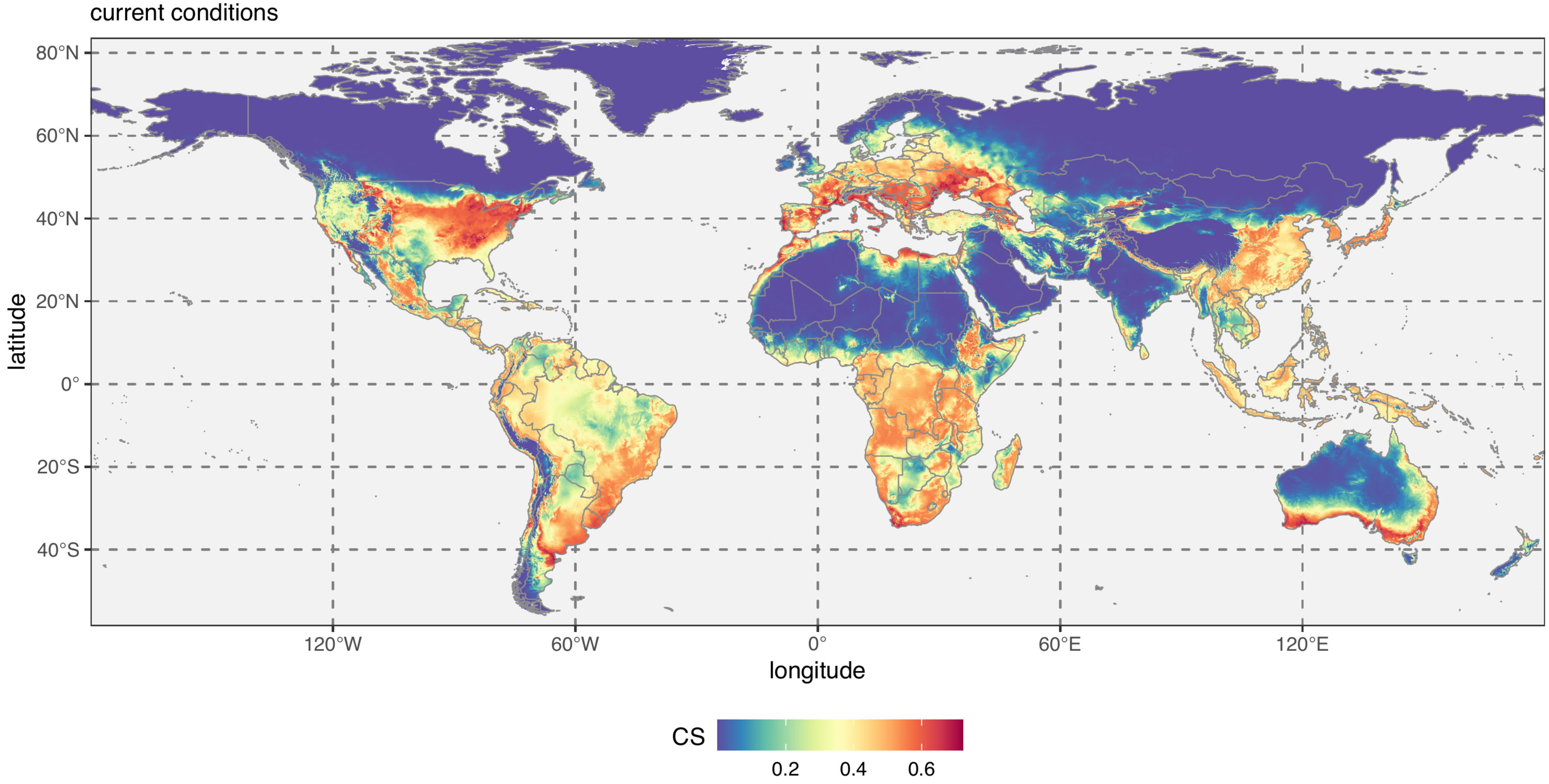

Figure S4: Potential geographical distribution of the brown marmorated stink bug *Halyomorpha halys* according to current climate conditions. The map depicts the climate suitability estimated using a Maxent model. Climate suitability varies from 0 (unsuitable) to 1 (maximum suitability). The maps were generated using R 4.0.3 (https://cran.r-project.org/).

DOI: 10.1038/s41598-021-90378-1

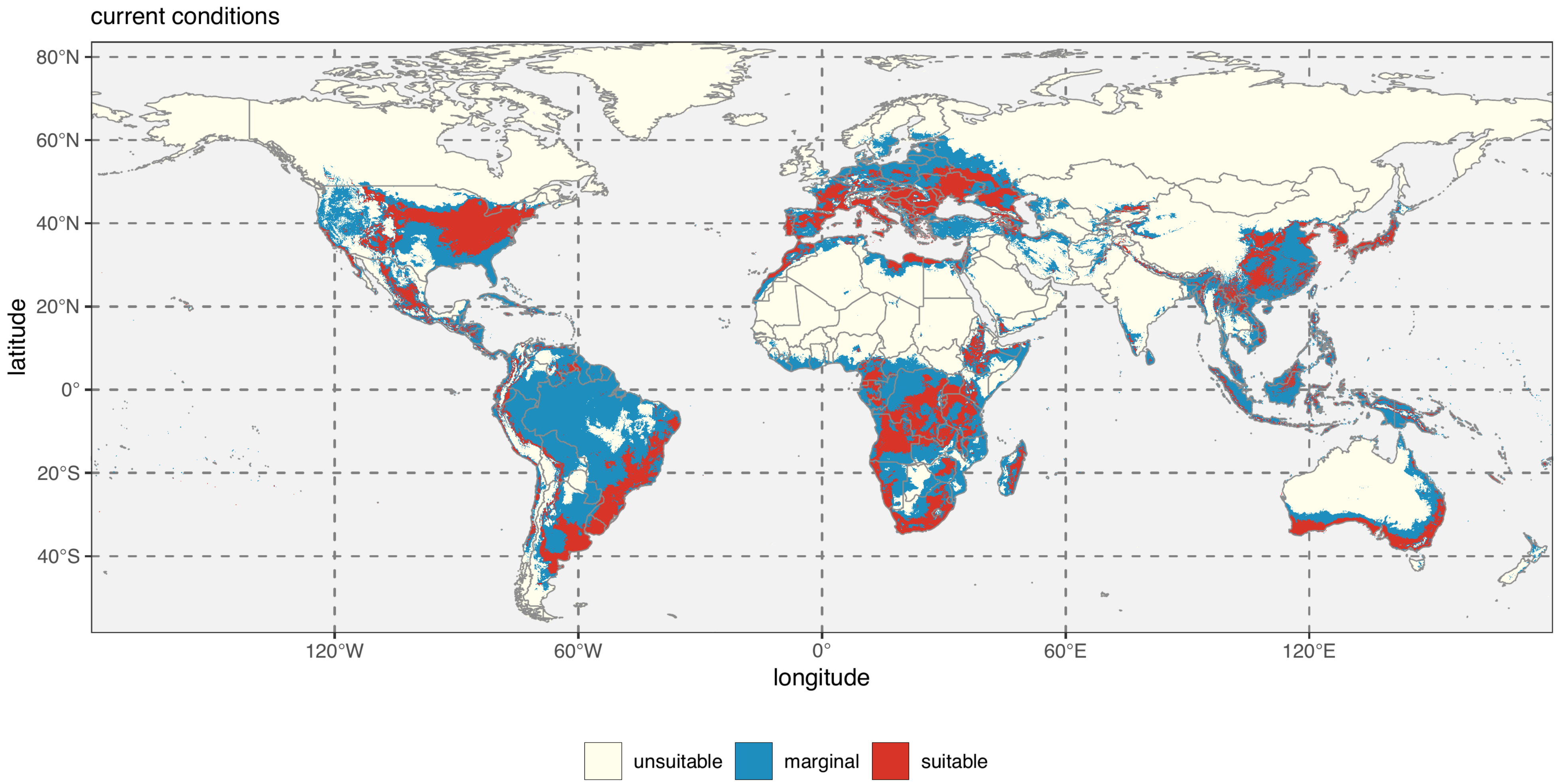

Figure S5: Potential geographical distribution of the brown marmorated stink bug *Halyomorpha halys* according to current climate conditions. The map depicts the climate suitability estimated using a Maxent model reclassified according to three classes : unsuitable, marginal and suitable. The maps were generated using R 4.0.3 (https://cran.r-project.org/).

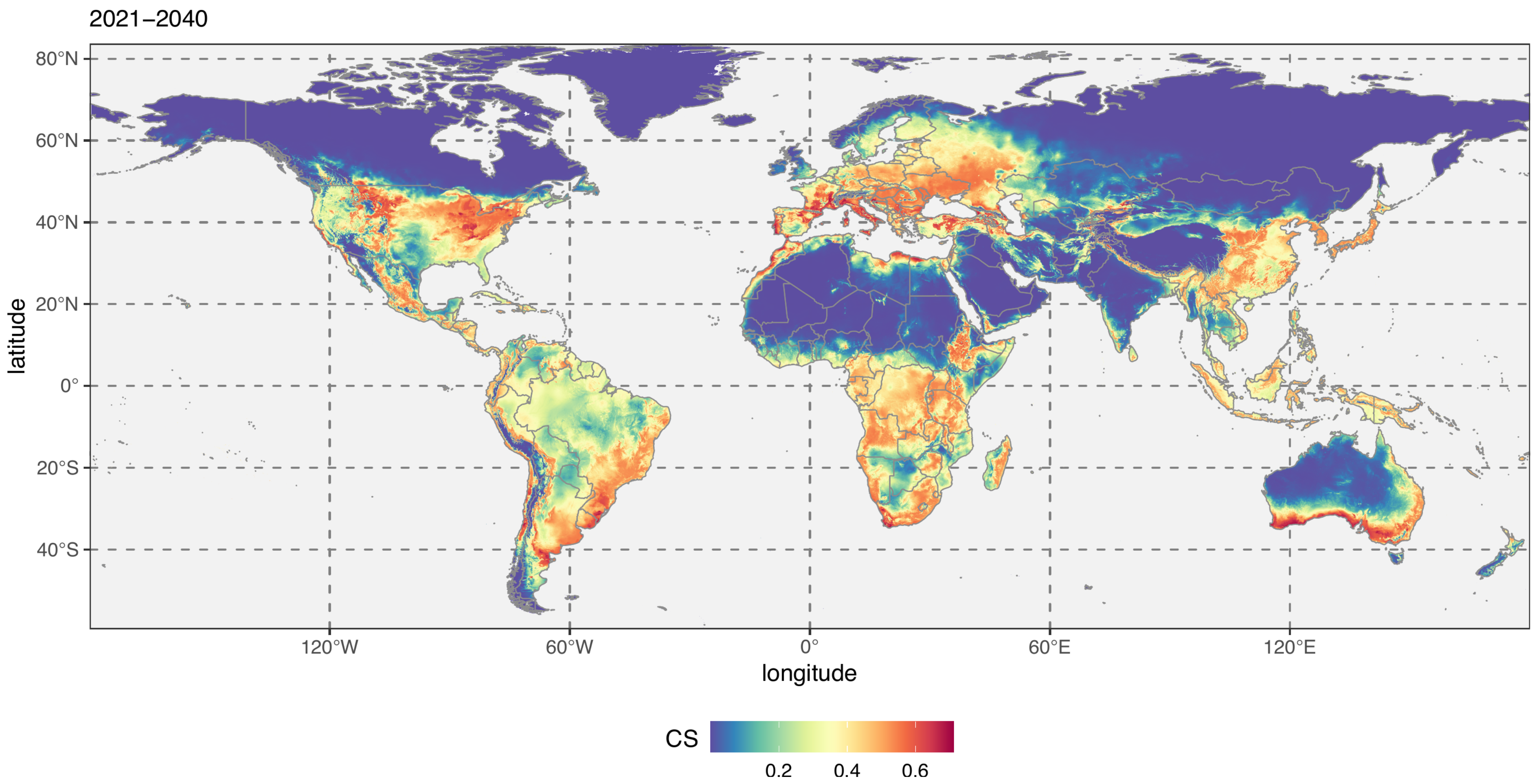

Figure S6: Potential geographical distribution of the brown marmorated stink bug *Halyomorpha halys* for the period 2021-2040 according to the future climate projections developed in the Coupled Model Intercomparison Project Phase 6 (CMIP6)(Eyring et al. 2016). The map depicts the median of a Maxent model projections according to 6 Global Circulation Models (GCM) for the shared socio-economic pathways SSP245. GCMs are BCC-CSM2-MR, CNRM-CM6-1, CNRM-ESM2-1, CanESM5, MIROC-ES2L and MIROC6. The Maxent model was calibrated using current conditions. The maps were generated using R 4.0.3 (https://cran.r-project.org/).

● Eyring et al (2016) Overview of the Coupled Model Intercomparison Project Phase 6 (CMIP6) experimental design and organization. Geosci Model Dev 9:1937-1958.

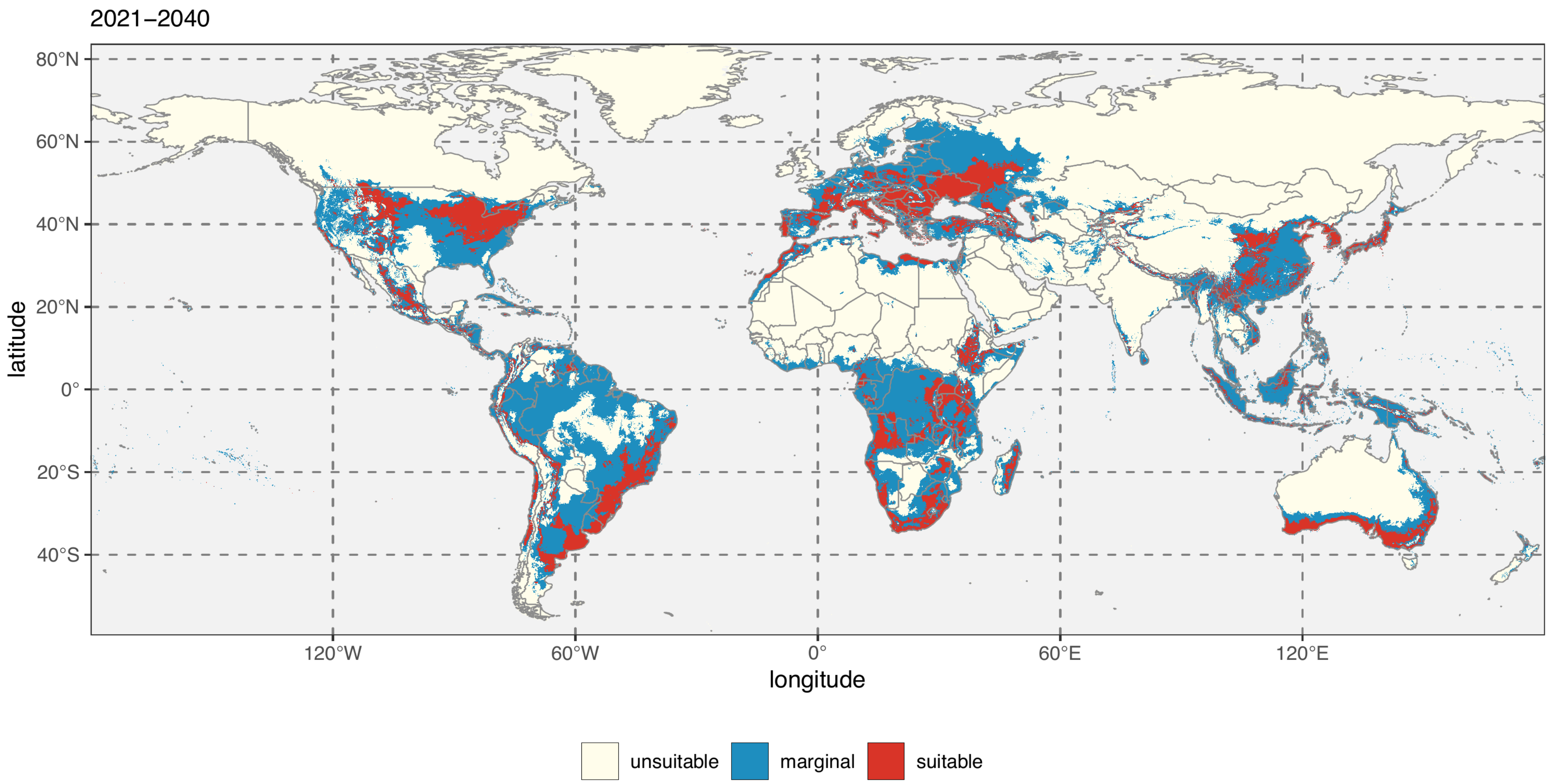

Figure S7: Potential geographical distribution of the brown marmorated stink bug *Halyomorpha halys* for the period 2021-2040 according to the future climate projections developed in the Coupled Model Intercomparison Project Phase 6 (CMIP6)(Eyring et al. 2016). The map depicts the median of a Maxent model projections according to 6 Global Circulation Models (GCM) for the shared socio-economic pathways SSP245. GCMs are BCC-CSM2-MR, CNRM-CM6-1, CNRM-ESM2-1, CanESM5, MIROC-ES2L and MIROC6. The Maxent model was calibrated using current conditions. Climate suitability was reclassified according to three classes : unsuitable, marginal and suitable. The maps were generated using R 4.0.3 (https://cran.r-project.org/).

● Eyring et al (2016) Overview of the Coupled Model Intercomparison Project Phase 6 (CMIP6) experimental design and organization. Geosci Model Dev 9:1937-1958.

DOI: 10.1038/s41598-021-90378-1

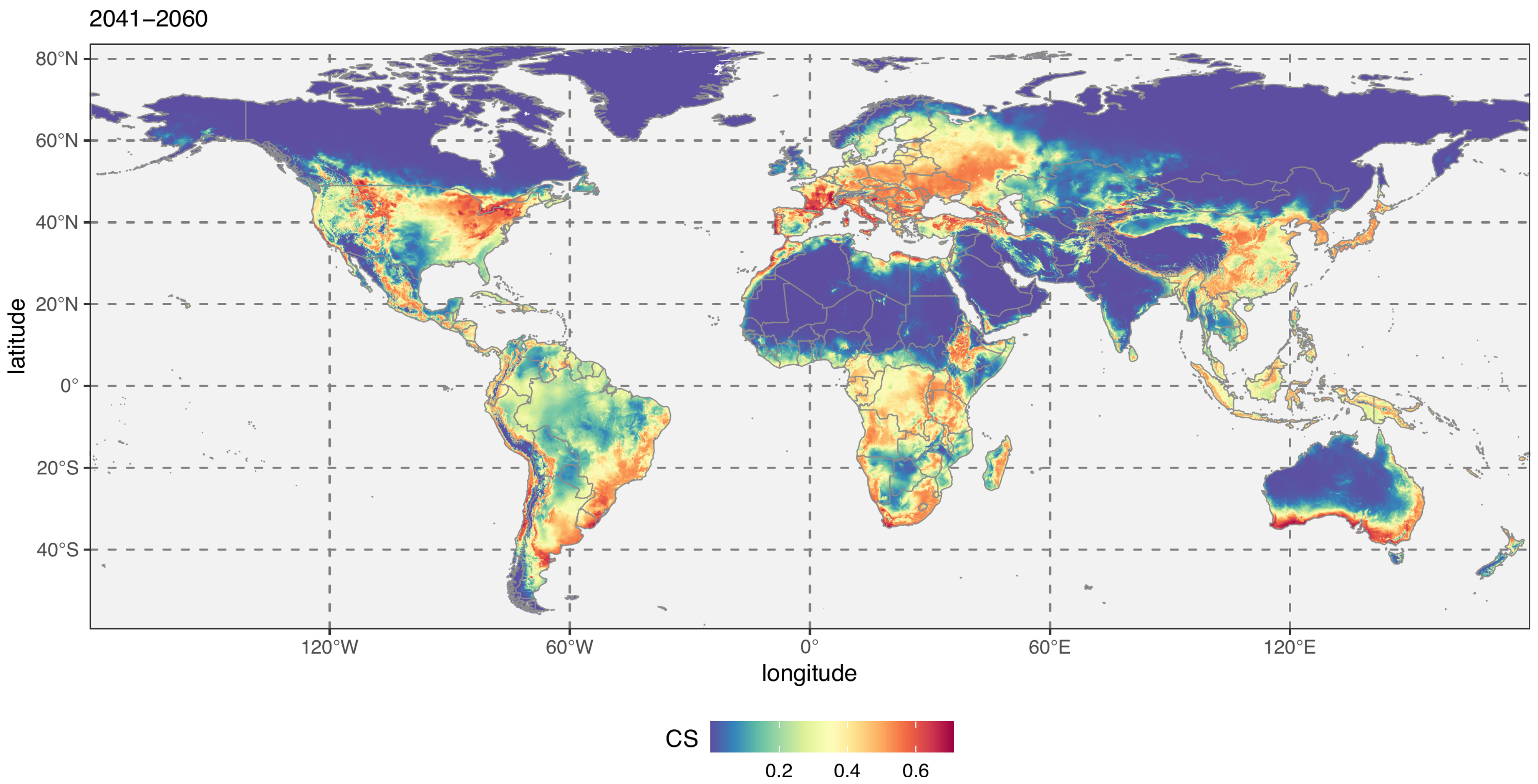

Figure S8: Potential geographical distribution of the brown marmorated stink bug *Halyomorpha halys* for the period 2041-2060 according to the future climate projections developed in the Coupled Model Intercomparison Project Phase 6 (CMIP6)(Eyring et al. 2016). The map depicts the median of a Maxent model projections according to 6 Global Circulation Models (GCM) for the shared socio-economic pathways SSP245. GCMs are BCC-CSM2-MR, CNRM-CM6-1, CNRM-ESM2-1, CanESM5, MIROC-ES2L and MIROC6. The Maxent model was calibrated using current conditions. The maps were generated using R 4.0.3 (https://cran.r-project.org/).

● Eyring et al (2016) Overview of the Coupled Model Intercomparison Project Phase 6 (CMIP6) experimental design and organization. Geosci Model Dev 9:1937-1958.

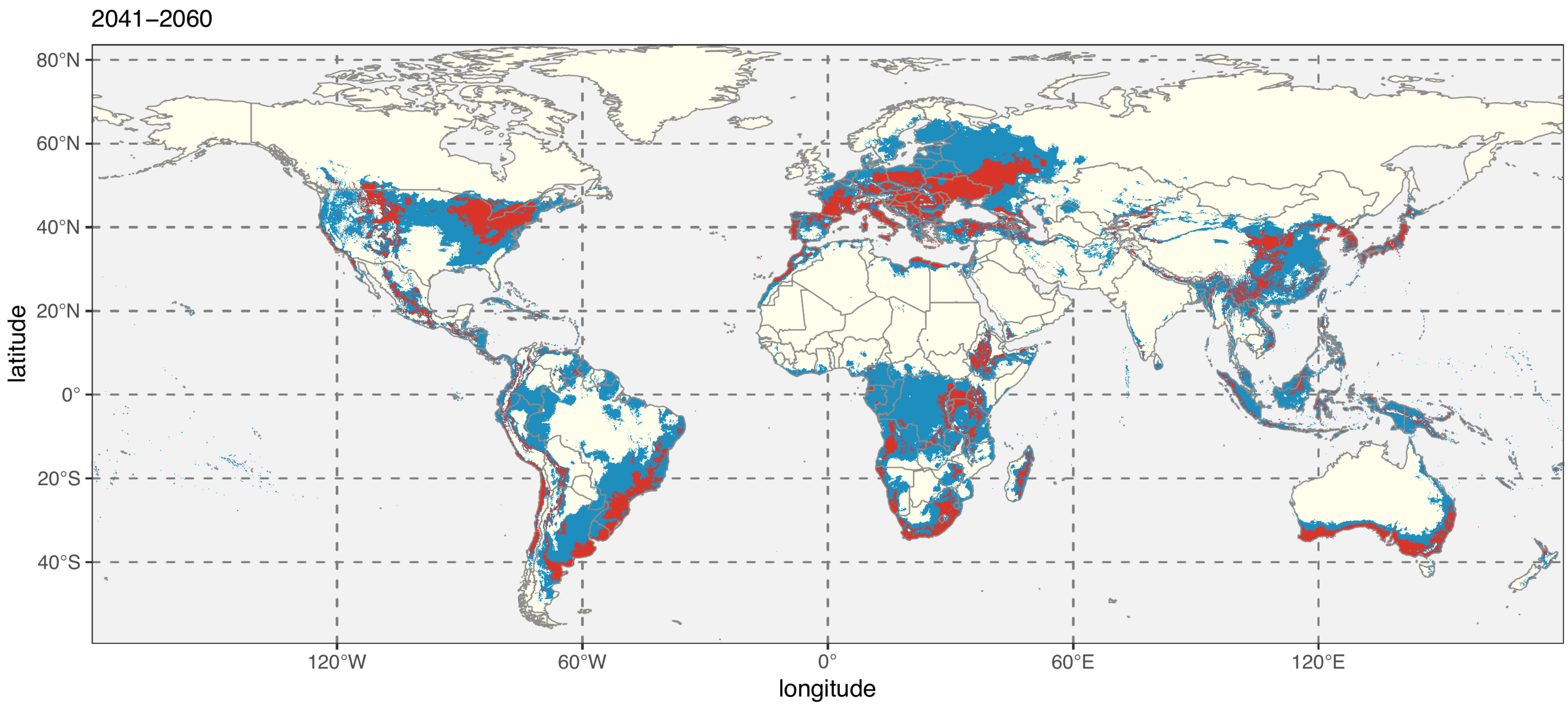

Figure S9: Potential geographical distribution of the brown marmorated stink bug *Halyomorpha halys* for the period 2041-2060 according to the future climate projections developed in the Coupled Model Intercomparison Project Phase 6 (CMIP6)(Eyring et al. 2016). The map depicts the median of a Maxent model projections according to 6 Global Circulation Models (GCM) for the shared socio-economic pathways SSP245. GCMs are BCC-CSM2-MR, CNRM-CM6-1, CNRM-ESM2-1, CanESM5, MIROC-ES2L and MIROC6. The Maxent model was calibrated using current conditions. Climate suitability was reclassified according to three classes : unsuitable, marginal and suitable. The maps were generated using R 4.0.3 (https://cran.r-project.org/).

● Eyring et al (2016) Overview of the Coupled Model Intercomparison Project Phase 6 (CMIP6) experimental design and organization. Geosci Model Dev 9:1937-1958.

DOI: 10.1038/s41598-021-90378-1
